# Impact of tapering targeted therapies (bDMARDs or JAKis) on the risk of serious infections and adverse events of special interest in patients with rheumatoid arthritis or spondyloarthritis: a systematic analysis of the literature and meta-analysis

**DOI:** 10.1186/s13075-020-02188-x

**Published:** 2020-04-29

**Authors:** D. Vinson, L. Molet-Benhamou, Y. Degboé, A. den Broeder, F. Ibrahim, C. Pontes, R. Westhovens, J. Závada, T. Pham, T. Barnetche, A. Constantin, A. Ruyssen-Witrand

**Affiliations:** 1 Rheumatology Department, CHU Sainte Marguerite, 270 Boulevard de Sainte-Marguerite, 13009 Marseille, France; 2 Rheumatology Department, Hôpital Purpan, Toulouse, France; 3 INSERM U1043, CPTP, Toulouse, France; 4 Department of Rheumatology, Sint Maartenskliniek, 6500 GM Nijmegen, The Netherlands; 5 Centre for Rheumatic Diseases, Department of Inflammation Biology, Faculty of Life Sciences and Medicine, School of Immunology and Microbial Sciences, King’s College London, Cutcombe Road, London, SE5 9RJ UK; 6 Department of Pharmacology, Therapeutics and Toxicology, Unitat docent Parc Taulí—Medical School—Universitat Autònoma de Barcelona, Sabadell, Barcelona Spain; 7 Skeletal Biology and Engineering Research Center, Department of Development and Regeneration, Rheumatology University Hospitals Leuven, KULeuven, Leuven, Belgium; 8 First Faculty of Medicine, Institute of Rheumatology and Department of Rheumatology, Charles University, Na Slupi 4, Prague, Czech Republic; 9 Rheumatology Department, Pellegrin Hospital, Bordeaux, France

**Keywords:** Rheumatoid arthritis, Spondyloarthritis, Biological therapies, DMARDs, Infection bacteria, Viruses infection, Systematic review

## Abstract

**Objectives:**

To systematically review the impact of tapering targeted therapies (bDMARDs or JAKis) on the risk of serious infections and severe adverse events (SAEs) in patients with rheumatoid arthritis (RA) or axial spondyloarthritis (axSpA) in remission or low disease activity (LDA) state.

**Materials and methods:**

A meta-analysis based on a systematic review of PubMed, Embase, Cochrane, until August 2019, as well as relevant databases of international conferences, was used to evaluate the risk difference (RD) at 95% confidence interval (95% CI) of incidence density of serious infections, SAEs, malignancies, cardiovascular adverse events (CV AEs), or deaths after tapering (dose reduction or spacing) compared to continuation of targeted therapies.

**Results:**

Of the 1957 studies initially identified, 13 controlled trials (9 RA and 4 SpA trials) were included in the meta-analysis. 1174 patient-years were studied in the tapering group (TG) versus 1086 in the usual care group (UC). There were 1.7/100 patient-year (p-y) serious infections in TG versus 2.6/100 p-y in UC (RD (95% CI) 0.01 (0.00 to 0.02), *p* = 0.13) and 7.4/100 p-y SAEs in TG versus 6.7/100 p-y in UC (RD 0.00 (− 0.02 to 0.02), *p* = 0.82). The risk of malignancies, CV AEs, or deaths did not differ between the tapering and the usual care groups. Subgroup analysis (RA and SpA) detected no significant differences between the two groups.

**Conclusion:**

We could not show significant impact of tapering bDMARD or JAKi over continuation concerning the risk of serious infections, SAEs, malignancies, CV AEs, or deaths in RA and SpA patients in remission or LDA state.

## Key message

What is already known about this subject?

► Current guidelines recommend tapering of targeted therapies for RA or SpA patients in remission.

What does this study add?

► No change in serious infection risk, SAEs, CV AEs, malignancies while tapering targeted therapies.

How might this impact on clinical practice?

► Physicians should still try to taper targeted therapies for efficiency and financial advantages.

## Introduction

Current best practice in the treatment of chronic inflammatory rheumatisms such as rheumatoid arthritis (RA) and spondyloarthritis (SpA) focusses on a tight control strategy (treat-to-target strategy) to prevent joint destruction and improve patients’ functional prognosis. The objective of this management strategy is to establish remission or a low disease activity (LDA) ((a) RA: Disease Activity Score28 ESR (DAS28-ESR) < 3.2 for LDA and < 2.6 for remission [1], (b) axSpA: Ankylosing Spondylitis Disease Activity Score (ASDAS) < 2.1 for LDA and < 1.3 for inactive disease [2]).

The first-line treatment for RA consists of conventional synthetic disease-modifying anti-rheumatic drugs (csDMARDs) [3], whereas initial treatment for SpA is based on non-steroidal anti-inflammatory drugs (NSAIDs) [4, 5]. Thereafter, in both diseases, biological DMARDs (bDMARDS) are employed, and the use of bDMARDs and more recently targeted synthetic DMARDs (tsDMARDs) has been part of the clear improvement in the therapeutic management of these diseases, leading to sustained LDA or remission in a large number of patients [6, 7].

Nonetheless, many studies have drawn attention to the increased AE risk occurring as a result of these treatments compared to csDMARD or no treatment, of which infectious disorders [8–10] are the most frequently reported. Patients on bDMARDs do not have an increased risk of malignancies in general [11, 12], but the risk of melanoma may be slightly increased. As for cardiovascular AE (CV AE), it is well-established [13, 14] that certain targeted therapies help improve cardiovascular comorbidities. The economic burden of these expensive therapies [15] should also not be underestimated and ought to be taken into consideration by the prescribing physician. The European League Against Rheumatisms (EULAR) and the American College of Rheumatology (ACR) have addressed these issues by publishing recommendations for the management of chronic inflammatory rheumatisms once remission or LDA has been established, based on a tapering strategy [3–5, 16–18].

Several studies [19–22] have highlighted that discontinuation of targeted therapies, more so in RA than in SpA, leads to an increased risk of relapse and radiographic progression, while tapering strategies does not seem to increase this risk when compared with continuing the initial treatment.

Although there is extensive evidence to support that tapering of targeted therapies does not significantly increase the risk of relapse nor of radiographic progression, a beneficial effect on the rate of infectious AEs, and most importantly on serious infectious, is yet to be proven. A recent meta-analysis [23] reassessed the effectiveness of down-titration compared with the continuation of the standard dose of anti-TNF for RA treatment in patients with LDA. Their study evaluated safety events as a secondary outcome and concluded that it was uncertain whether anti-TNF tapering influenced the number of SAE observed due to the very low certainty of the evidence obtained. This study was restricted to RA patients treated with anti-TNF and did not extend to SpA patients or any other targeted therapies.

The aim of our study was therefore to assess the impact of tapering targeted therapies (bDMARDs or JAKis), compared to continuation of the initial treatment regimen, on the risk of serious infectious and AEs of special interest (SAEs, malignancies, CV AEs, and deaths) in patients with RA or SpA, in remission or LDA, by conducting a systematic analysis of the literature and a meta-analysis.

## Materials and methods

This meta-analysis is reported in accordance with the Preferred Reporting Items for Systematic Review and Meta-Analysis Protocols (PRISMA-P) 2015 statement [24] (see Supplementary Text 1).

### Selection of articles

We carried out a systematic analysis of the literature to identify controlled trials, preferentially prospective and randomized trials, which compared tapering targeted therapies (bDMARDs or JAKis) versus continuation of the initial treatment regimen, in patients with RA or SpA in remission or LDA. The PubMed, Embase, and Cochrane databases were searched from their date of inception to August 2019 using a Boolean association of keywords (see Supplementary Text 2)***.*** Abstracts from articles submitted to international conferences (ACR, EULAR, and SFR) between 2016 and 2019 were also queried.

This search was carried out independently by two investigators (DV and LMB). The title and abstract of articles identified from database searches were subsequently reviewed for the following inclusion criteria: (1) controlled trials, randomized or not; (2) involving rheumatoid arthritis (RA) or spondyloarthritis (SpA) patients; (3) treated by targeted therapies: bDMARDs (anti-TNF (adalimumab, certolizumab, etanercept, golimumab, infliximab) or abatacept or anti-IL6 (sarilumab, tocilizumab) or rituximab or anti IL 12/23 (ustekinumab) or anti IL 17 (secukinumab, ixekizumab) or anti-IL 23) or JAKis (tofacitinib, baricitinib or upadacitinib); (4) in remission or LDA under targeted therapies; and (5) comparing tapering (dose reduction or spacing [3]) targeted therapies (tapering group (TG)) versus continuation of the initial treatment regimen (usual care group (UC)).

The other inclusion criteria applied after full text reading were (1) description of targeted therapies tapering protocol and (2) assessing at least one of the following AE: serious infections, SAEs, CV AEs, malignancies, or death. We did not include any restrictions concerning disease duration, period of remission or LDA, duration of treatment, or concomitant use of csDMARDs. The limits were English or French language. The exclusion criteria were (1) retrospective trials, (2) case reports, (3) trials without tapering of targeted therapies, (4) trials without control arms, and (5) trials with no data on AE.

### Data extraction

Data was collated using a standardized grid. For each selected study, predefined data were extracted (see Supplementary Text 3).

If data were missing in the article, the corresponding authors were contacted by e-mail. Details of data collected are available in Supplementary Table 1 and 2.

Patient and public involvement was not appropriate in our study.

### Study quality assessment

Risk of bias was assessed using the Cochrane Risk of Bias Tool [25] and is available in Supplementary Figure 1***.***

### Analyses

For the meta-analysis, the primary endpoint was the incidence density of serious infections in each treatment group (TG or UC). The secondary endpoints were the incidence density of SAEs, CV AEs, malignancies, or deaths.

A risk difference (RD) was calculated for each study included in the meta-analysis. All meta-analyses were performed using the inverse variance approach, which assumes a fixed effect model, to determine the weight given to each study. This provided a common weighted RD estimate with a 95% CI, taking into account weighting of the different samples. RD and 95% CIs are expressed as forest plots. Statistical heterogeneity of the selected studies was tested using the *Q* test (*χ*^2^), applying a 0.05 statistical significance cut-off, and reported with the *I*^2^ statistic in which high values of *I*^2^, ranging from 0 to 100%, represent strong heterogeneity. In case of a significant heterogeneity, a random effect model was applied to take into account heterogeneity. Publication bias was searched using funnel plots and Egger test, no significant bias was found (Supplementary Figure 2). All computations were performed using the RevMan V.5.3 software package developed by Nordic Cochrane Centre (Review Manager (computer program), V.5.3. Copenhagen: The Nordic Cochrane Centre, the Cochrane Collaboration, 2011). *P* values lower than 0.05 were considered significant.

## Results

### Study selection

**Fig. 1:**
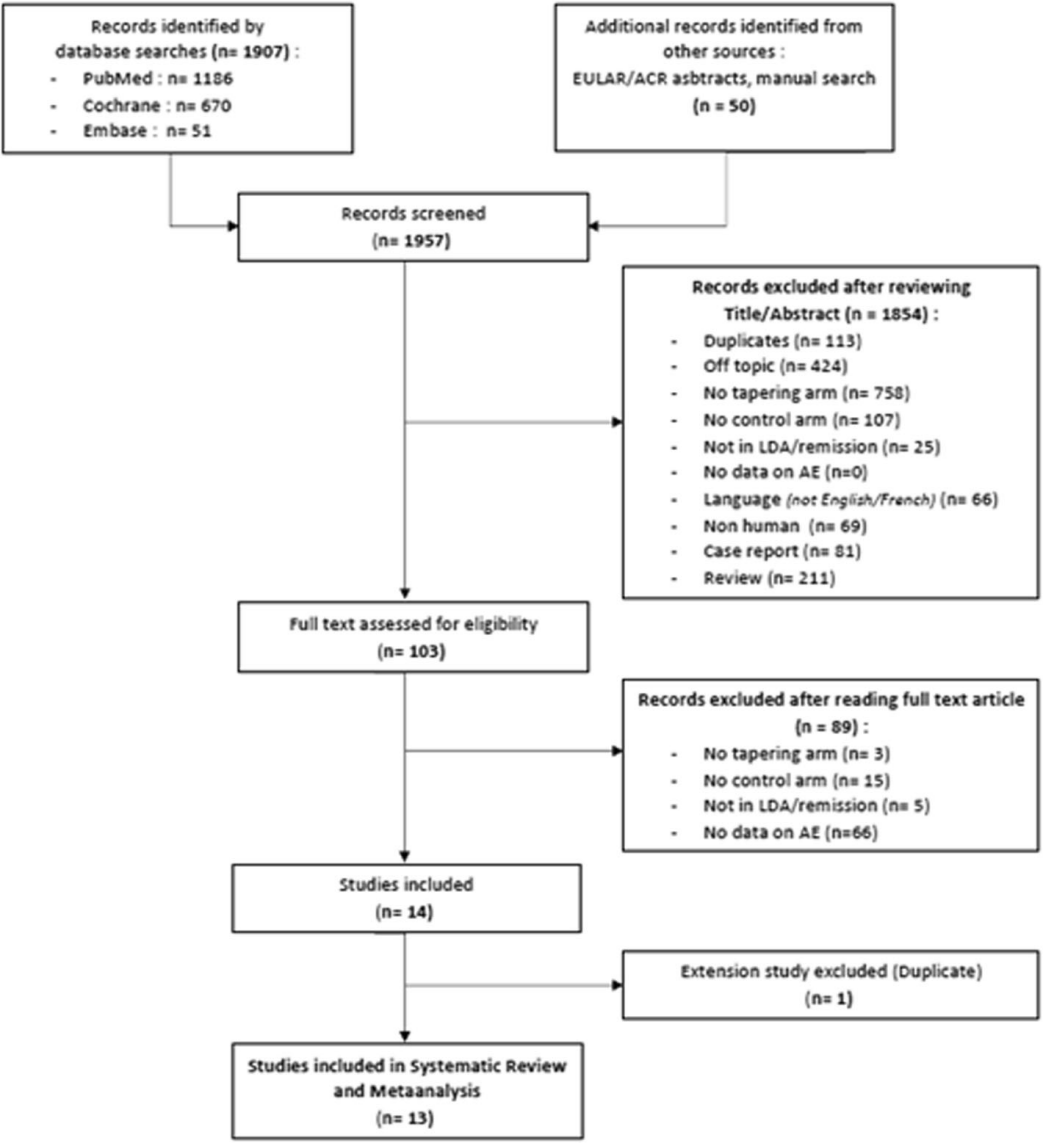
Flow chart of systematic review and meta-analysis

1957 records were screened in our systematic analysis of the literature. 1854 records were excluded based on title and abstract reading, leaving a total of 103 relevant articles to be further examined. Full-text assessments excluded an additional 89 references. Among the remaining 14 studies, one study [26], corresponding to an extension phase of another included study which already described AEs, was excluded in order to avoid duplicated data. Thirteen references were finally included in our systematic review and meta-analysis (Fig. 1).

We contacted 11 corresponding authors by e-mail to complete safety data that was omitted from the corresponding publications (values and details). Five authors [27–31] responded and were able to provide us with the necessary information to complete our data set.

### Population characteristics

Among the 13 studies included in the analysis, there were 9 RA trials [27, 28, 30, 32–37] and 4 SpA trials [29, 31, 38, 39], more precisely axial SpA. All were controlled trials, 11 were randomized controlled trials [26–28, 30–38], whereas 2 studies followed a longitudinal observational design using a propensity score matching method [29, 39].

A total of 2196 patients were included. Disease duration extended from 2.2 to 16.6 years, the sex ratio was 65% female, and mean patient age in both groups ranged from 30 to 59 years. Disease activity was low in both groups (TG and UC): DAS 28-CRP ranging from 1.6 to 2.3 in RA patients and BASDAI SpA patients from 1 to 2.

#### Duration of the trials

1174 patient-years were studied in the targeted therapies tapering group (TG) versus 1086 in the usual care group (UC). The study period extended from 6 months in 2 trials [28, 37] to 12 months in 8 trials [29–35, 39] and to approximately 18 months in 3 trials [27, 36, 38].

#### Studied drugs

In RA patients, the studied targeted therapies were predominantly anti-TNF: certolizumab [32], adalimumab [27, 28, 36, 37], and etanercept [27, 28, 34–36]. Abatacept, a selective modulator of T cell co-stimulation, was studied in one trial [30], and baricitinib, a targeted synthetic DMARD (JAK-inhibitor), was also evaluated in one trial [33]. SpA patient drug treatments were based on anti-TNF: adalimumab [29, 31], etanercept [29, 31, 38, 39], infliximab [29, 31], and golimumab [31].

#### Previous treatment and duration of targeted therapies

Patients were bDMARD-naive prior to their inclusion in 6 trials [27, 29, 30, 32, 35, 38], whereas they received bDMARDs before the investigation in 4 trials [31, 33, 36, 37]. This information was not available for 3 studies [28, 34, 39]. No study included patients treated with tsDMARDs prior to inclusion in the trials.

In bDMARD-naive patients, the duration of targeted therapies ranged from 37 weeks to 6 years, with a preponderance of patients taking targeted therapies for more than 3 years. For patients previously treated with bDMARDs prior to their inclusion in the trial, data relating to the total duration of the targeted therapy could not be retrieved.

### Evaluating the primary outcome: serious infections in studies comparing tapering of targeted therapies (TG) versus usual care (UC)

**Fig. 2:**
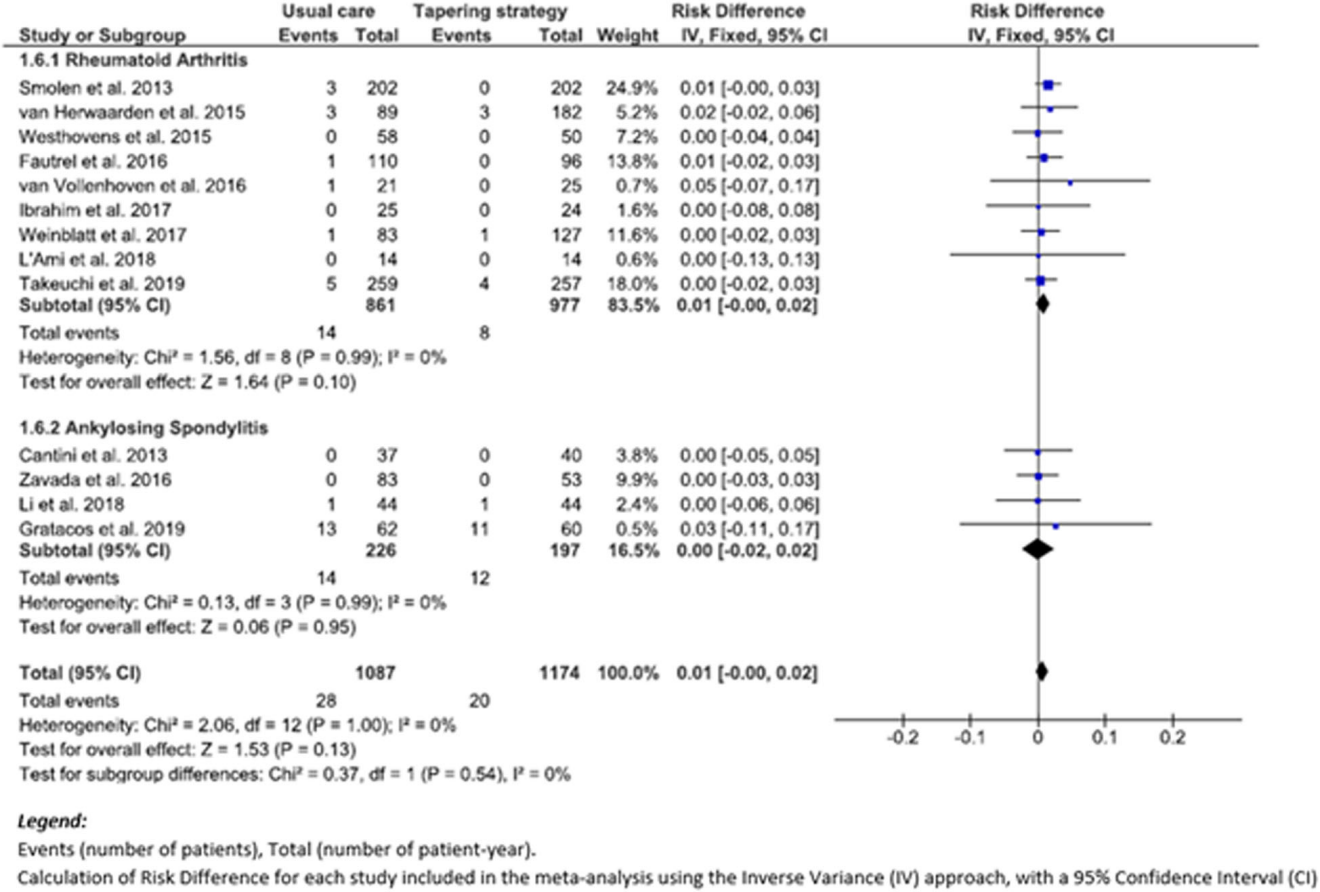
Forest plot of serious infections: difference of risk of serious infections in TG versus UC

Our meta-analysis comparing tapering of targeted therapies (TG) versus continuation of usual care (UC) was performed on 13 trials and showed no significant difference in relation to the incidence density of serious infections reported between TG (20 patients presented serious infections among 1174 patient-years, corresponding to 1.7/100 patient-year (p-y)) and UC groups (28 patients presented serious infections among 1087 patient-years (2.6/100 p-y)), with a total risk difference (RD) (95% CI) of 0.01 (0.00 to 0.02), *p* = 0.13 (*I*^2^ heterogeneity score, 0%) (Fig. 2).

The subgroup analysis, performed separately on RA or SpA trials, did not show any significant difference in the risk of serious infections in RA patients, 0.01 (0.00 to 0.02), *p* = 0.10 (*I*^2^, 0%), nor in SpA patients, 0.00 (− 0.02 to 0.02), *p* = 0.95 (*I*^2^, 0%) (Fig. 2).

### Evaluation of secondary outcomes: SAEs

**Fig. 3:**
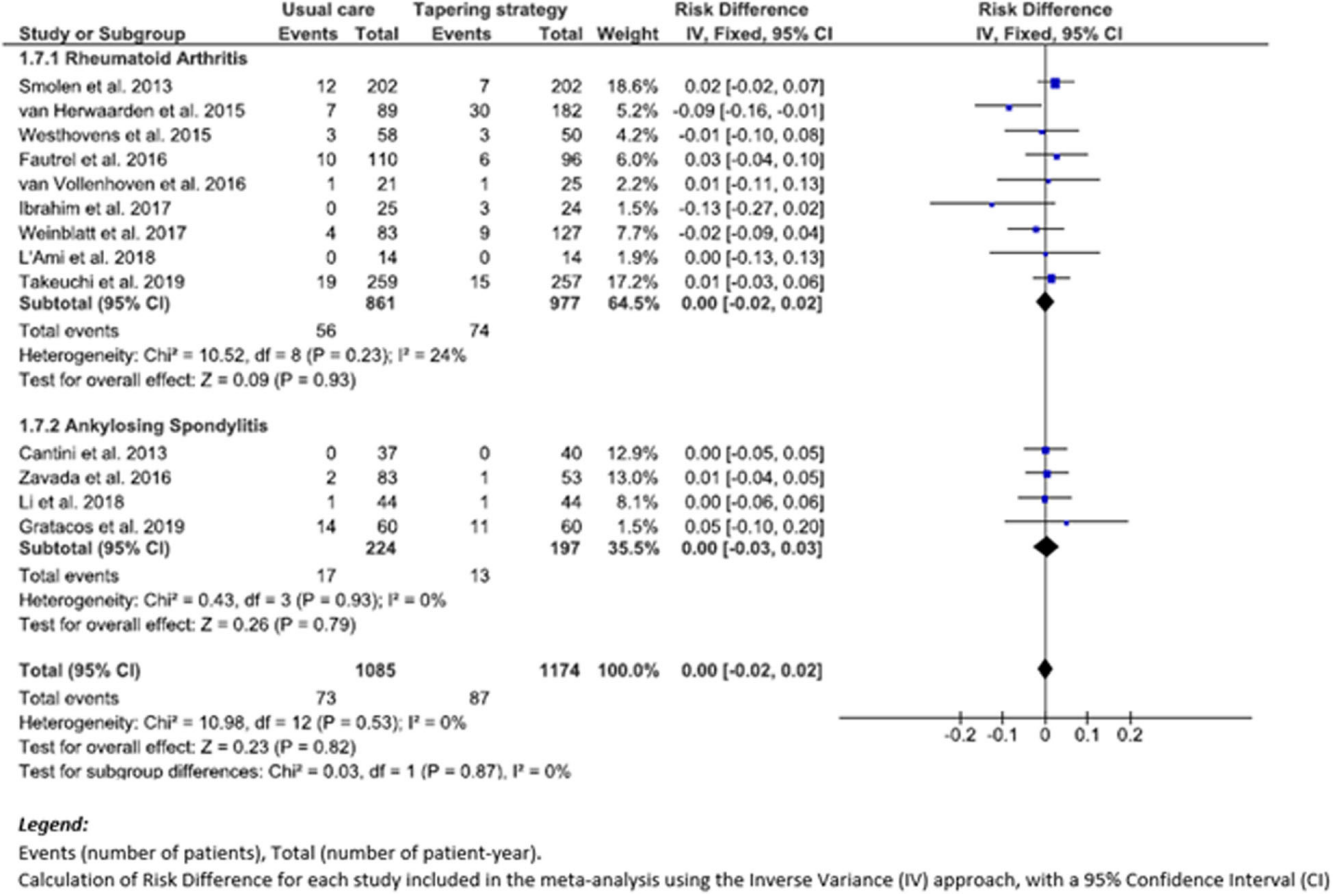
Forest plot of severe adverse events: risk difference of TG versus UC

Our meta-analysis did not show a decreased risk in SAEs (RD (95% CI), 0.00 (− 0.02 to 0.02), *p* = 0.82 (*I*^2^, 0%)), when comparing 87 patients with SAEs among 1174 patient-years (7.4/100 p-y) in the TG group to 73 patients with SAEs among 1085 patient-years (6.7/100 p-y) in the UC group (Fig. 3). There was also no statistically significant difference between the two subgroups: RD in RA patients (95% CI) was 0.00 (− 0.02 to 0.02), *p* = 0.93 (*I*^2^, 24%), compared to RD in SpA patients (95% CI) 0.00 (− 0.03 to 0.03), *p* = 0.79 (*I*^2^, 0%) (Fig. 3).

### Meta-analysis of other safety events in studies comparing TG versus UC

**Fig. 4:**
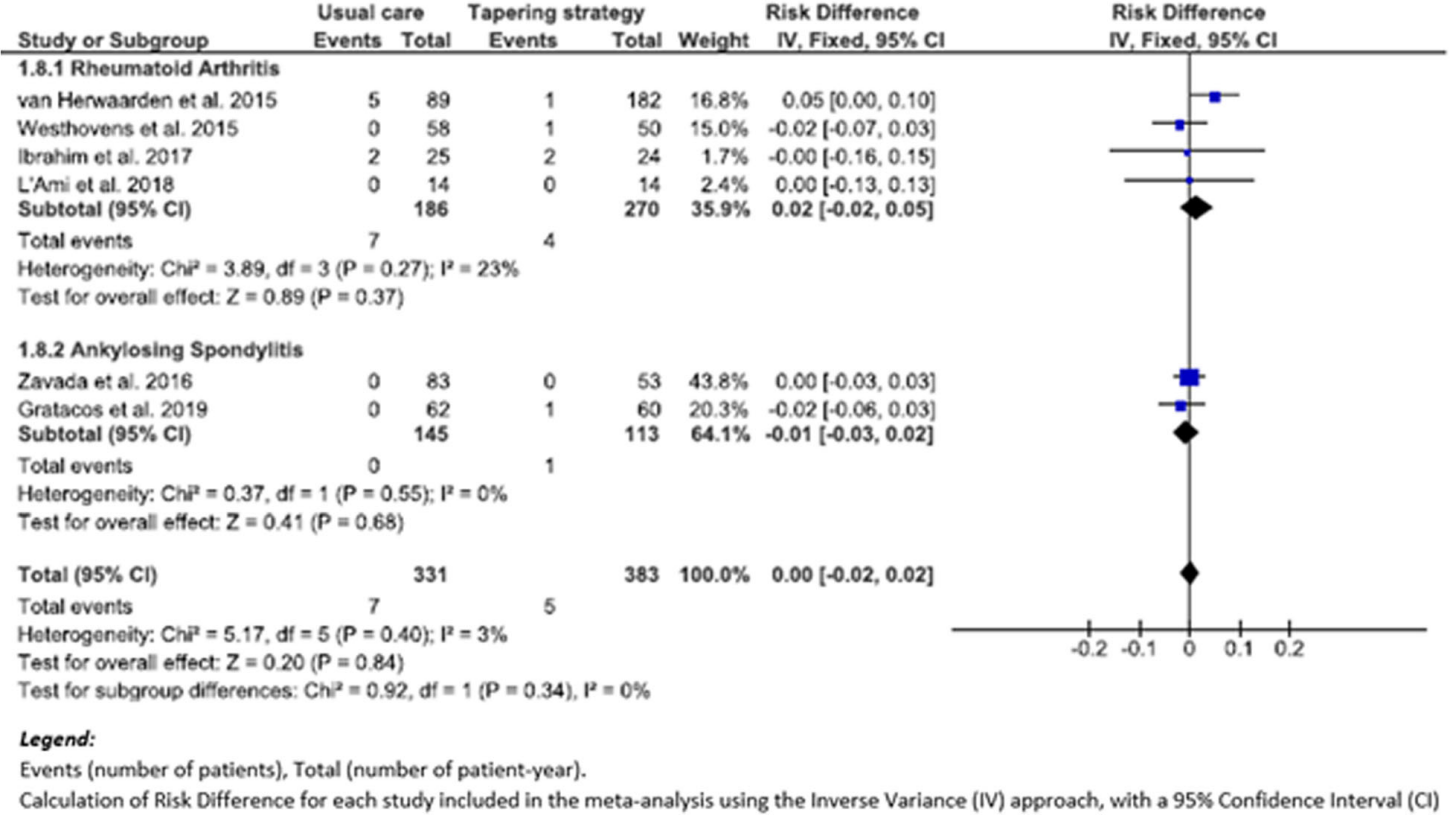
Forest plot of cardiovascular adverse events: risk difference of TG versus UC

Six studies [27–31, 37] reported the incidence of CV AEs. Among these, 4 studies [27, 28, 30, 37] focused on RA patients and 2 studies [29, 31] on SpA patients. There was no significant difference in the incidence of CV AE (RD (95% CI), 0.00 (− 0.02 to 0.02), *p* = 0.84 (*I*^2^, 3%)), when comparing 5 patients with CV AE among 383 patients-years (1.3/100 p-y) in TG, to 7 patients with CV AE among 331 patients-years (2.1/100 p-y) in UC (Fig. 4). There was no significant difference in each subgroup either: in RA patients, RD (95% CI) was 0.02 (− 0.02 to 0.05), *p* = 0.37 (*I*^2^, 23%), whereas in SpA patients RD (95% CI) was − 0.01 (− 0.03 to 0.02), *p* = 0.68 (*I*^2^, 0%) (Fig. 4).

**Fig. 5:**
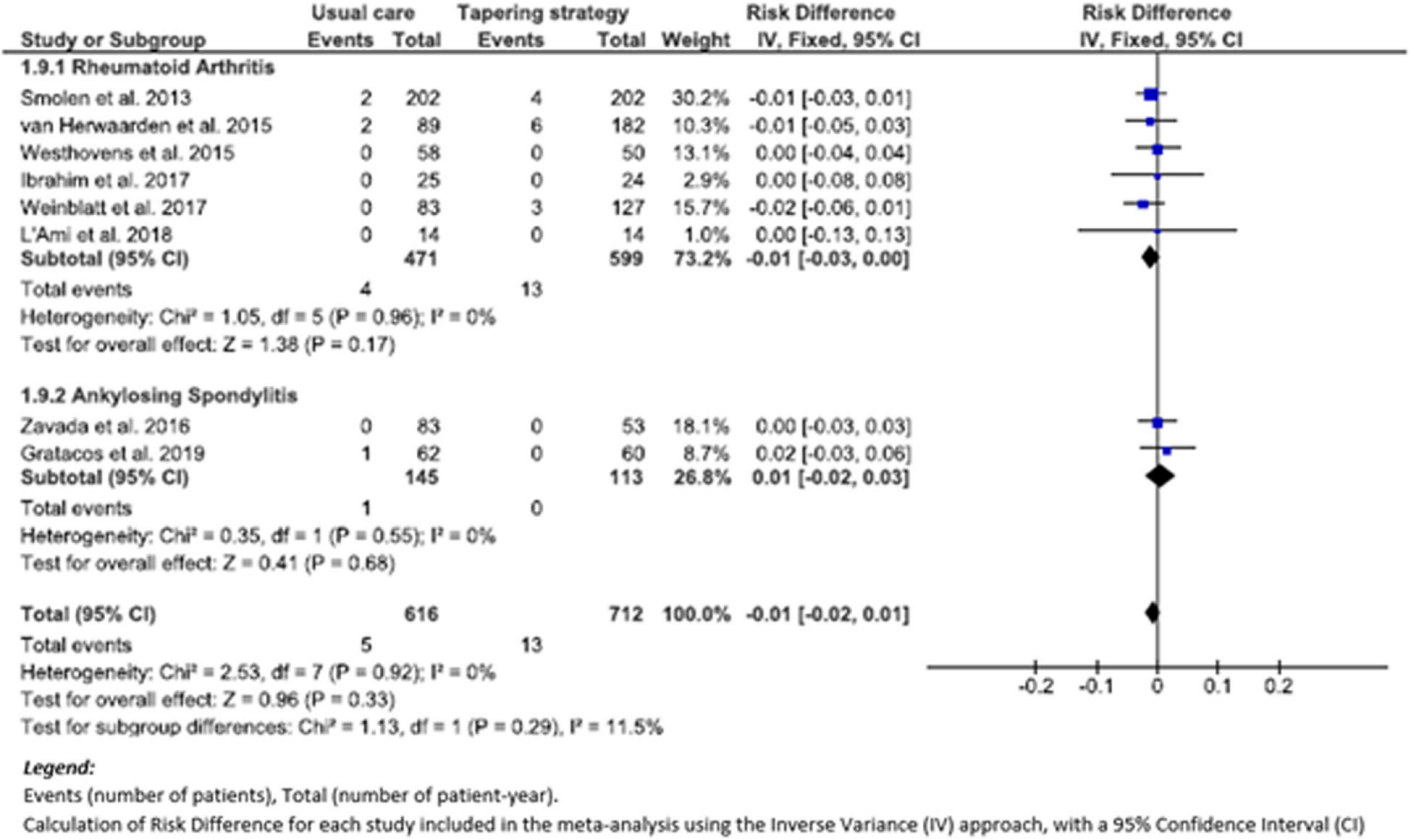
Forest Plot of Malignancy: Risk difference of TG versus UC

Eight studies [27–32, 35, 37] reported malignancies which developed during the study period. Among these, 6 studies [27, 28, 30, 32, 35, 37] involved RA patients and 2 studies [29, 31] SpA patients; 13 patients with malignancies among 712 patients-years (1.8/100 p-y) in TG and 5 patients with malignancies among 616 patients-years (0.8/100 p-y) in UC. Our meta-analysis did not detect any significant differences when all 8 studies were examined (RD (95% CI), − 0.01 (− 0.02 to 0.01), *p* = 0.33 (*I*^2^, 0)) and also not in the subgroup analysis (RA patients: RD (95% CI), − 0.01 (− 0.03 to 0.00), *p* = 0.17 (*I*^2^, 0%); SpA patients: RD (95% CI), 0.01 (− 0.02 to 0.03), *p* = 0.68, (*I*^2^, 0%)) (Fig. 5).

**Fig. 6:**
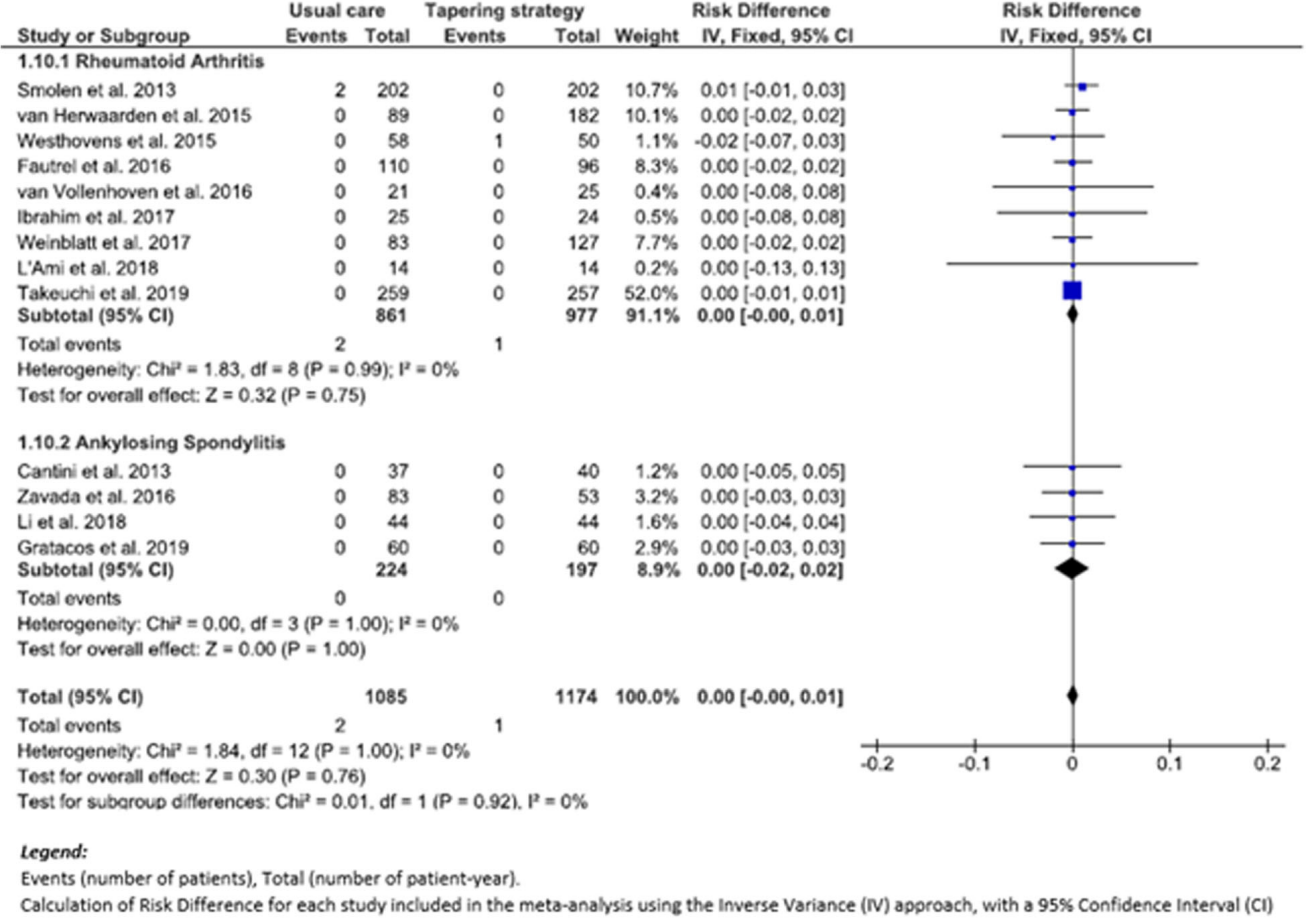
Forest Plot of Deaths: Risk difference of TG versus UC

Three deaths were reported in the trials, 2 in one RA trial [35] in the UC group (0.2/100 p-y) and 1 in one RA trial (0.1/100 p-y) in the TG group [30], with no significant differences detected (RD (95% CI), 0.00 (0.00 to 0.01), *p* = 0.76 (*I*^2^, 0%)) (Fig. 6).

## Discussion

Our meta-analysis focused on controlled trials in RA and SpA patients that achieved remission or LDA and showed that tapering targeted therapies was not associated with a statistically significant difference in risk of developing serious infections, SAEs, CV AEs, malignancies, or death when compared with continuation of the initial treatment regimen. This result was also confirmed in the subgroup analyses (of RA and SpA). Our results are consistent with the only other meta-analysis [23] published on the issue.

Our meta-analysis has several strengths. Compared to the previously published meta-analysis, it includes SpA patients next to RA patients and expands on the number and type of drugs used (bDMARDS and JAKis) and finally assesses specific subgroups of AE outcomes. Also, all studies included in our meta-analysis were prospective, controlled trials, and eleven [27, 28, 30–38] out of thirteen studies randomly assigned patients into groups, thereby granting the absence of heterogeneity.

We expected to see a decrease of the risk of serious infections due to a reduction of the therapeutic pressure. However, although we observed a numerical trend with less serious infections in tapering versus continuation of the initial treatment schedule (1.7/100 p-y versus 2.6/100 p-y), our study was not able to show a statistical difference. The range of serious infections observed in our meta-analysis is consistent with a previous one focused on bDMARDs in RA patients [10]. A recent meta-analysis [23] published in 2019, which focused on down-titration compared with continuation of the standard dose of TNFi for RA in patients with LDA, evaluated safety events as secondary outcomes and concluded that it was uncertain whether dose tapering affected the number of SAEs observed due to the low certainty of the evidence. Another meta-analysis [10] assessing the risk of serious infections in RA patients treated with targeted therapies was conducted in 2015 and also did not detect any differences. Indeed, the initial course of treatment with targeted therapies may represent an increased risk of infectious events [40]. However, the absence of statistical difference may be explained by a lack of power. Indeed, we calculated the statistical power of our meta-analysis, based on the primary endpoint (serious infections), which was about 26.6%. This is relatively low and can be explained by the assessed endpoint being rare and by the fact that the clinical trials included in our meta-analysis were not designed to achieve an objective of tolerance. To increase the power, it would require a greater number of patients and would jeopardize the feasibility of these types of trials. Indeed, to reach a statistical power of 80% in our meta-analysis, it would require 10,084 patient-year. In addition, the difference in frequency is very low between the two groups (TG vs UC) for each tolerance endpoints, and this also contributes to the requirement for greater patient numbers in order to show a significant difference. Despite its good internal validity, our meta-analysis has several limitations.

Only one study analyzing a tsDMARD (Baricitinib) was included in our meta-analysis, which makes the evaluation of infectious risk in this therapeutic subclass precarious, even though the results were consistent with a non-significant difference.

Another concern is the healthy survivor bias. After excluding subjects with numerous comorbidities, which may potentially influence the risk of infection [41, 42], most of all in RA patients, this risk may be lowered. Accordingly, it may become more difficult to point out a difference between patient populations that are already closely followed for any signs of AEs, during a longer follow-up and with better tolerance. Moreover, patients in these trials had been taking targeted therapies for at least more than a year, before initiating the tapering phase. However, the critical phase for AE is in the first months [43–45]. Indeed, a safety issue (serious infection and SAE) is more likely to happen in the initiating phase, rather than after a long period under treatment, thus leading to the exclusion of the patient. The population included in the trials is therefore highly selected and might explain the absence of any difference in risk between the TG and UC group.

The same argument holds for the selection of subjects regarding cardiovascular and tumoral status.

As for CV AE, it is well-established [13, 14] that certain targeted therapies help improve cardiovascular comorbidities in patients, yet no increase in CV AE was detected in the TG strategy. This may potentially be explained by the persistence of the remission/LDA state, imparting lower cardiovascular risk [46].

Regarding malignancy, the duration of the studies was too short and the events too rare to detect any statistical difference. Trials with a longer follow-up period would be needed to be able to point out a statistical difference.

## Conclusion

In conclusion, our meta-analysis highlights no remarkable difference in the rate of infectious events, in patients in the tapering treatment group or patients continuing their initial treatment schedule.

Nevertheless, other benefits of a tapering strategy, including alleviating patient burden due to self-injection, medication costs, and the safety of these strategies in relation to flare-ups leads us to support this therapeutic approach.

## Supplementary information

**Additional file 1 : Supplementary text 1**: Preferred Reporting Items for Systematic review and Meta-Analysis Protocols (PRISMA-P) 2015 statement [24]. **Supplementary text 2**: Boolean association of keywords used in PubMed (Medline database). **Supplementary text 3**: List of data extracted for each selected study. **Supplementary Table 1**: Population characteristics of studies included in the Meta-Analysis. **Supplementary Table 2**: Adverse Event (AE) characteristics of studies included in the Meta-Analysis. **Supplementary Figure 1**: Cochrane Risk of Bias Tool [25]. **Supplementary Figure 2**: Publication bias and Funnel plots.

## Data Availability

This study involved data that are available in published papers used for this systematic review. Additional data were also got directly from authors.

## Abbreviations

ACR: American College of Rheumatology
ASDAS: Ankylosing Spondylitis Disease Activity Score
axSpA: Axial spondyloarthritis
CV AE: Cardiovascular adverse events
DAS28-ESR: Disease Activity Score28 ESR
DMARDs: Disease-modifying anti-rheumatic drugs
bDMARDs: Biological DMARDs
csDMARDs: Conventional synthetic disease-modifying anti-rheumatic drugs
tsDMARDs: Targeted synthetic DMARDs
EULAR: European League Against Rheumatisms
JAKi: JAKinibs
LDA: Low disease activity
NSAIDs: Non-steroidal anti-inflammatory drugs
p-y: Patient-years
RA: Rheumatoid arthritis
RD: Risk difference
SAE: Severe adverse events
TG: Tapering group
UC: Usual care group
