# Supplementary material for: Impact of tapering targeted therapies (bDMARDs or JAKis) on the risk of serious infections and adverse events of special interest in patients with rheumatoid arthritis or spondyloarthritis: a systematic analysis of the literature and meta-analysis
Source: Arthritis Res Ther. 2020 Apr 29;22:97. doi: 10.1186/s13075-020-02188-x (PMC7191828; doi:10.1186/s13075-020-02188-x)
Supplement: Supplementary file 1 — Additional file 1 : Supplementary text 1: Preferred Reporting Items for Systematic review and Meta-Analysis Protocols (PRISMA-P) 2015 statement [24]. Supplementary text 2: Boolean association of keywords used in PubMed (Medline database). Supplementary text 3: List of data extracted for each selected study. Supplementary Table 1: Population characteristics of studies included in the Meta-Analysis. Supplementary Table 2: Adverse Event (AE) characteristics of studies included in the Meta-Analysis. Supplementary Figure 1: Cochrane Risk of Bias Tool [25]. Supplementary Figure 2: Publication bias and Funnel plots. [file 13075_2020_2188_MOESM1_ESM.docx]

**SUPPLEMENTARY MATERIALS**

**Supplementary text 1: Preferred Reporting Items for Systematic review and Meta-Analysis Protocols (PRISMA-P) 2015 statement** (24)

| **Section/topic** | **#** | **Checklist item** | **Information reported** | | **Line number(s)** |
| --- | --- | --- | --- | --- | --- |
|  |  |  | **Yes** | **No** |  |
| **ADMINISTRATIVE INFORMATION** | | | | | |
| **Title** | | | | | |
| Identification | 1a | Identify the report as a protocol of a systematic review |  |  | Cover page |
| Update | 1b | If the protocol is for an update of a previous systematic review, identify as such |  |  | Cover page |
| **Registration** | 2 | If registered, provide the name of the registry (e.g., PROSPERO) and registration number in the Abstract |  |  | Not done |
| **Authors** | | | | | |
| Contact | 3a | Provide name, institutional affiliation, and e-mail address of all protocol authors; provide physical mailing address of corresponding author |  |  | Cover page |
| Contributions | 3b | Describe contributions of protocol authors and identify the guarantor of the review |  |  | Cover page |
| **Amendments** | 4 | If the protocol represents an amendment of a previously completed or published protocol, identify as such and list changes; otherwise, state plan for documenting important protocol amendments |  |  | None |
| **Support** | | | | | |
| Sources | 5a | Indicate sources of financial or other support for the review |  |  | None |
| Sponsor | 5b | Provide name for the review funder and/or sponsor |  |  | None |
| Role of sponsor/funder | 5c | Describe roles of funder(s), sponsor(s), and/or institution(s), if any, in developing the protocol |  |  | None |
| **INTRODUCTION** | | | | | |
| **Rationale** | 6 | Describe the rationale for the review in the context of what is already known |  |  | p3-4 |
| **Objectives** | 7 | Provide an explicit statement of the question(s) the review will address with reference to participants, interventions, comparators, and outcomes (PICO) |  |  | p3-4 |
| **METHODS** | | | | | |
| **Eligibility criteria** | 8 | Specify the study characteristics (e.g., PICO, study design, setting, time frame) and report characteristics (e.g., years considered, language, publication status) to be used as criteria for eligibility for the review |  |  | p5-6 |
| **Information sources** | 9 | Describe all intended information sources (e.g., electronic databases, contact with study authors, trial registers, or other grey literature sources) with planned dates of coverage |  |  | 5-6 |
| **Search strategy** | 10 | Present draft of search strategy to be used for at least one electronic database, including planned limits, such that it could be repeated |  |  | p5-6 and Supplementary Text 2 |
| ***STUDY RECORDS*** | | | | | |
| Data management | 11a | Describe the mechanism(s) that will be used to manage records and data throughout the review |  |  | p5-6 |
| Selection process | 11b | State the process that will be used for selecting studies (e.g., two independent reviewers) through each phase of the review (i.e., screening, eligibility, and inclusion in meta-analysis) |  |  | p5-6 |
| Data collection process | 11c | Describe planned method of extracting data from reports (e.g., piloting forms, done independently, in duplicate), any processes for obtaining and confirming data from investigators |  |  | p5-6 |
| **Data items** | 12 | List and define all variables for which data will be sought (e.g., PICO items, funding sources), any pre-planned data assumptions and simplifications |  |  | p5-6 |
| **Outcomes and prioritization** | 13 | List and define all outcomes for which data will be sought, including prioritization of main and additional outcomes, with rationale |  |  | p5-6 |
| **Risk of bias in individual studies** | 14 | Describe anticipated methods for assessing risk of bias of individual studies, including whether this will be done at the outcome or study level, or both; state how this information will be used in data synthesis |  |  | p6 and Supplementary Figure 1 |
| ***DATA*** | | | | | |
| **Synthesis** | 15a | Describe criteria under which study data will be quantitatively synthesized |  |  | p5-7 |
|  | 15b | If data are appropriate for quantitative synthesis, describe planned summary measures, methods of handling data, and methods of combining data from studies, including any planned exploration of consistency (e.g., *I* ^2^, Kendall’s tau) |  |  | p7, 10-14 |
|  | 15c | Describe any proposed additional analyses (e.g., sensitivity or subgroup analyses, meta-regression) |  |  | p7, 10-14 |
|  | 15d | If quantitative synthesis is not appropriate, describe the type of summary planned |  |  | NA |
| **Meta-bias (es)** | 16 | Specify any planned assessment of meta-bias(es) (e.g., publication bias across studies, selective reporting within studies) |  |  | p15-17 |
| **Confidence in cumulative evidence** | 17 | Describe how the strength of the body of evidence will be assessed (e.g., GRADE) |  |  | N/A |

**Supplementary text 2: Boolean association of keywords used in PubMed (Medline database)**

(Tapering OR reduction OR lowering OR decreasing OR de-escalation OR Lessening OR diminution OR deprescription OR spacing)

*AND*

(bDMARD* OR "anti IL 6" OR "IL 6 blocker" OR "IL 6 inhibitor" OR “anti interleukin 6” OR Sarilumab OR Tocilizumab OR anti-TNF OR "anti-tumor necrosis factor" OR "TNF inhibitor" OR "tumor necrosis factor inhibitor" OR "tumor necrosis factor blocker" OR "TNF blocker" OR "Tumor Necrosis Factor antagonist" OR “TNF antagonist” OR Abatacept OR Adalimumab OR Certolizumab OR Etanercept OR Golimumab OR Infliximab OR Rituximab OR “Janus Kinase Inhibitors” OR “JAK Inhibitors” OR "JAKinib" OR Tofacitinib OR Baricitinib OR Upadacitinib OR “Anti IL 12/23” OR Ustekinumab OR "anti IL 17" OR "IL 17 blocker" OR "IL 17 inhibitor" OR "anti interleukin 17” OR "interleukin 17 inhibitor" OR Secukinumab OR Ixekizumab OR "anti IL 23" OR "IL 23 inhibitor" OR "anti interleukin 23”)

*AND*

("Rheumatoid arthritis" OR "psoriatic arthritis" OR spondylarthritis OR "spondylitis ankylosing" OR spondyl* OR "Arthritis reactive" OR spondylarthropathies OR spondyloarthritis OR spondylarthropathy OR "arthritis, psoriatic" [MeSH Terms])

**Supplementary text 3: List of data extracted for each selected study**

- Reference: Authors, Name of article, Journal, Name of publication, Name of study.
- Study design/ characteristics: study design, mono/multicentre, geographic origin, trial duration.
- Population characteristics: type of rheumatism (RA/SpA), inclusion criteria, number of patients in each arm, disease duration, age, gender ratio, BMI, diagnosis criteria, initial activity score (BASDAI/DAS28-CRP), disease characteristics
- Initial treatment characteristic: targeted therapies, initial posology, duration under current targeted therapies, ratio of associated treatment (csDMARDs, corticoids, NSAIs).
- Interventions: treatment arm (tapering group (TG) or usual care (UC))
- Outcomes: number of patients with Serious Infections/ SAEs/ CV AEs/ Malignancies/ Death in each arm (TG or UC) during the study period.

**Supplementary Table 1: Population characteristics of studies included in the Meta-Analysis :**


**Supplementary Table 2: Adverse Event (AE) characteristics of studies included in the Meta-Analysis :**

**Supplementary Figure 1 : Cochrane Risk of Bias Tool** (25)


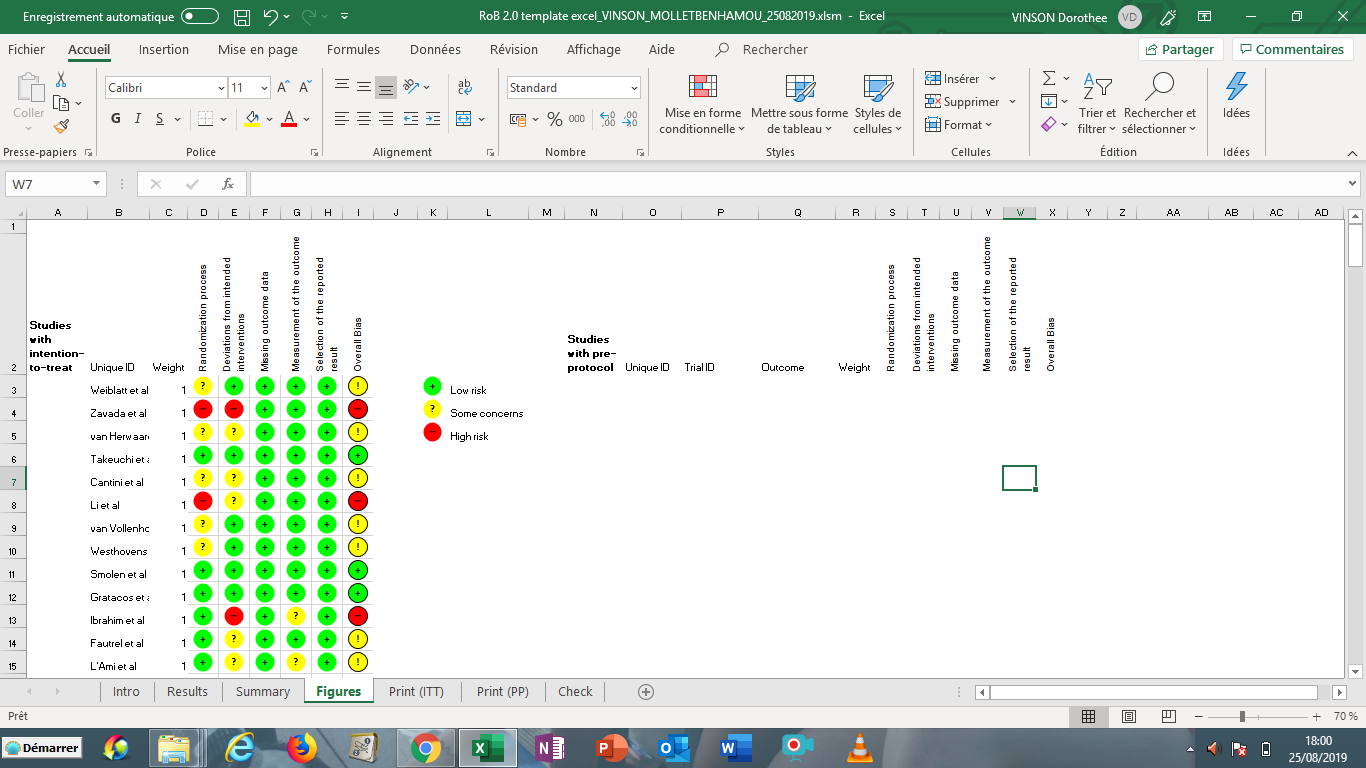


**Supplementary Figure 2: Publication bias and Funnel plots**

1.
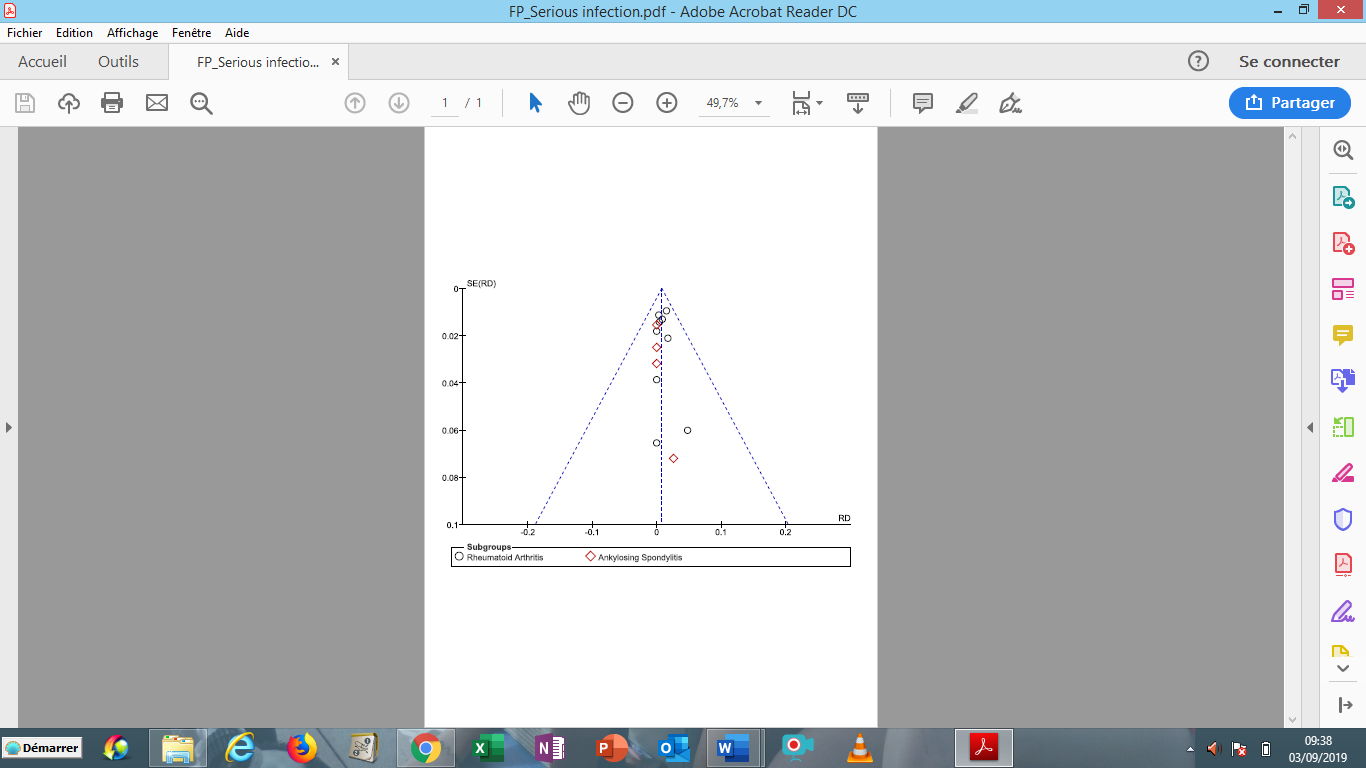
***Funnel plot: Serious infection***
2. ***Funnel plot: Severe Adverse Event (SAE)***


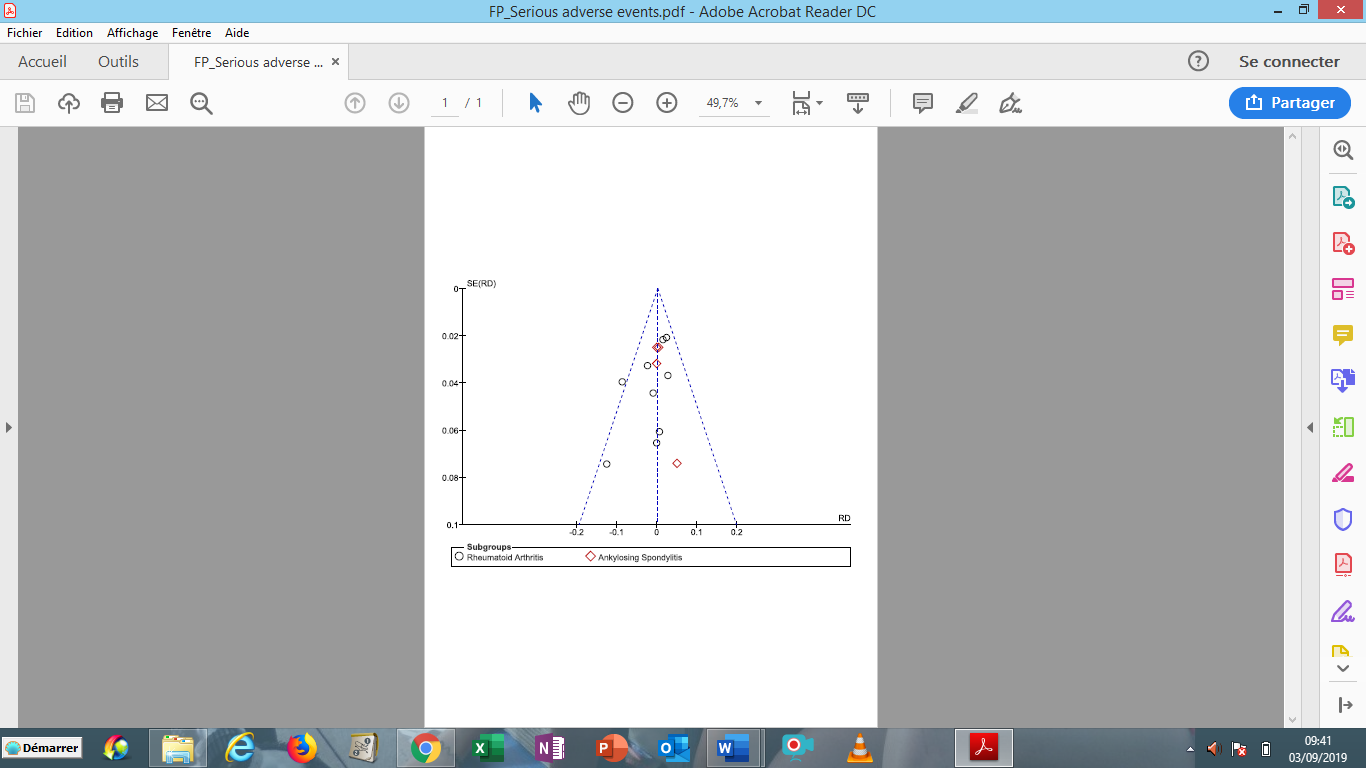


1.
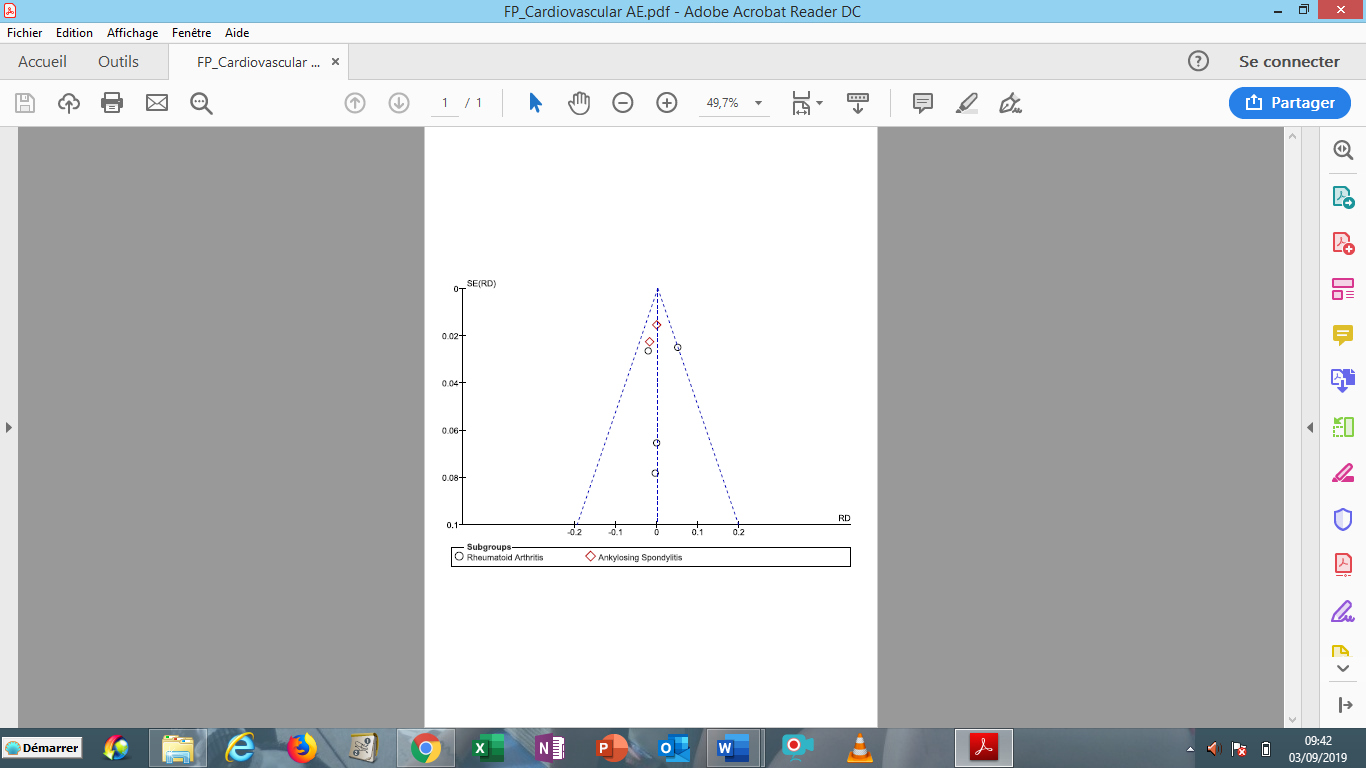
***Funnel plot: Cardiovascular Adverse Event (CV AE)***
2.
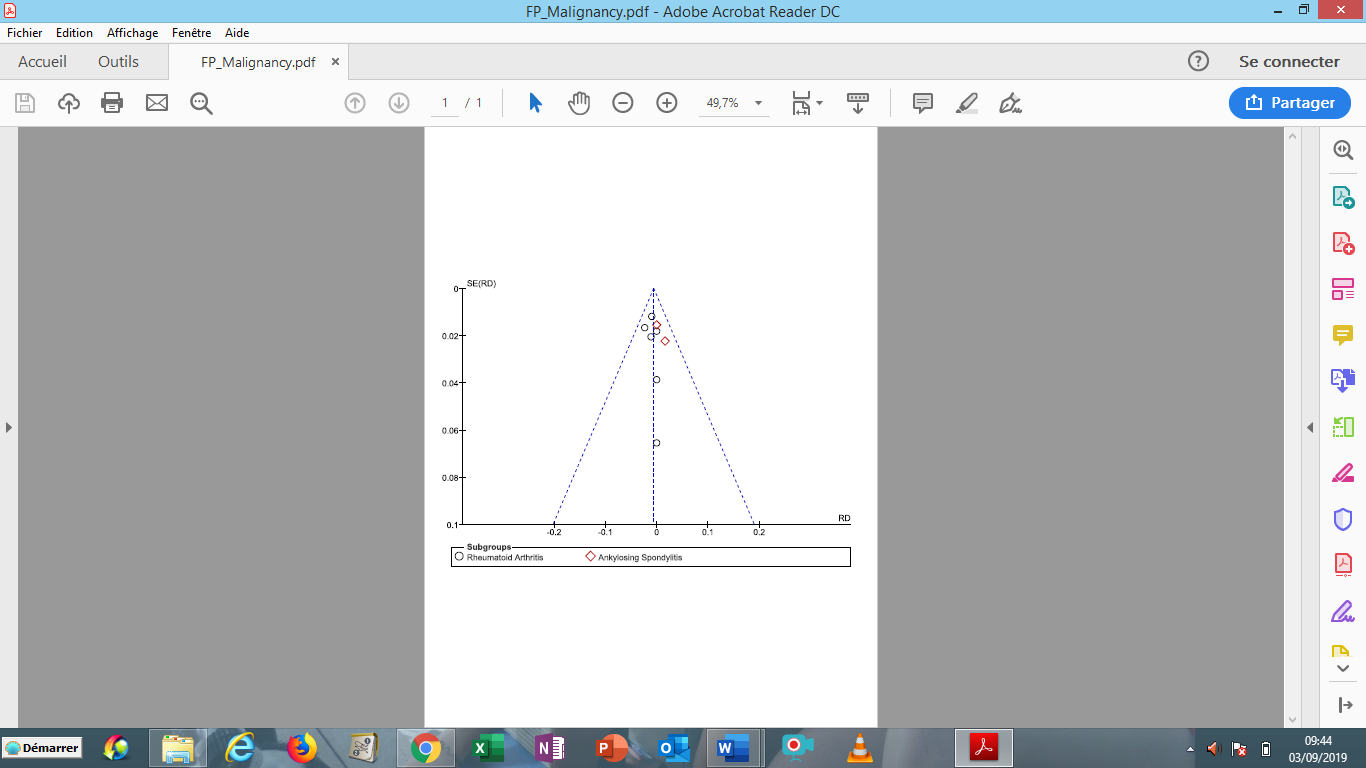
***Funnel plot: Malignancy***
3.
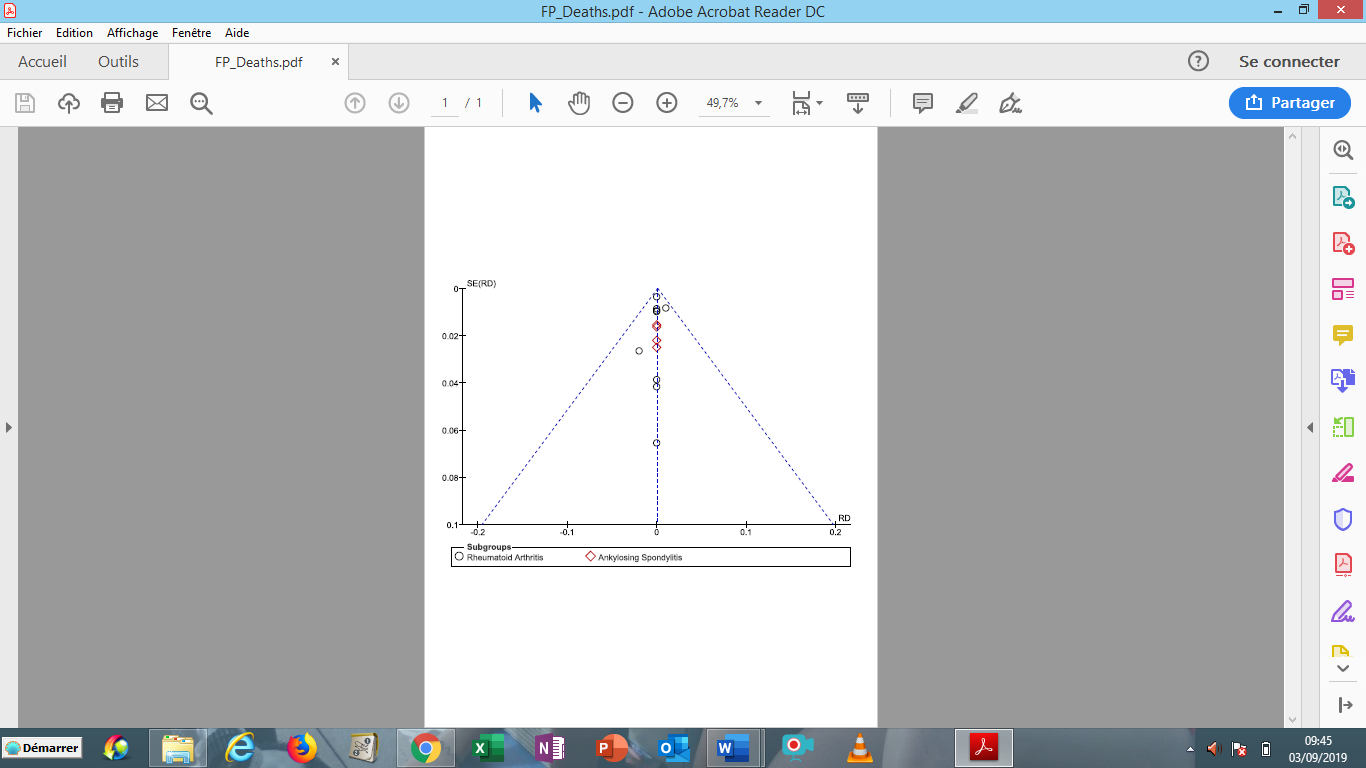
***Funnel plot : Deaths***
